# Correction: Examining acculturative stress among international students in Ghana using an interpretative phenomenological approach: Unpacking the social support systems

**DOI:** 10.1371/journal.pone.0322964

**Published:** 2025-04-22

**Authors:** Kwasi Gyasi-Gyamerah, Joseph Osafo, Angela Anarfi Gyasi-Gyamerah, Evans Sakyi Boadu

## Notice of republication

This article was republished on March 20, 2025, to correct an error in the author list: Kwasi Gyasi-Gyamerah was erroneously displayed as Kwasi-Gyasi Gyamerah and Joseph Osafo was erroneously displayed as Prof Joseph Osafo. Please download this article again to view the correct version. The originally published, uncorrected article and the republished, corrected article are provided here for reference.

## Supporting information

S1 File Originally published, uncorrected article. (PDF)

S2 File Republished, corrected article. (PDF)
