# Supplementary material for: Correction: Examining acculturative stress among international students in Ghana using an interpretative phenomenological approach: Unpacking the social support systems
Source: PLoS One. 2025 Apr 22;20(4):e0322964. doi: 10.1371/journal.pone.0322964 (PMC12013897; doi:10.1371/journal.pone.0322964)
Supplement: S1 File — (PDF) [file pone.0322964.s001.pdf]

PLOS ONE

RESEARCH ARTICLE

Examining acculturative stress among international students in Ghana using an interpretative phenomenological approach. Unpacking the social support systems

Kwasi-Gyasi Gyamerah[1], **Prof Joseph Osafo**[2], **Angela Anarfi Gyasi-Gyamerah**[2]*, **Evans Sakyi Boadu**[3,4]

1 Council on International Educational Exchange (CIEE), University of Ghana, Legon, Ghana, **2** Department of Psychology, University of Ghana, Legon, Ghana, **3** School of Governance, University of the Witwatersrand, Johannesburg, South Africa, **4** School of Sustainable Development, University of Environment and Sustainable Development (UESD), Somanya, Ghana

* aagyasi-gyamerah@ug.edu.gh

Check for updates

OPEN ACCESS

Citation: Gyamerah K-G, Osafo PJ, Gyasi-Gyamerah AA, Boadu ES (2024) Examining acculturative stress among international students in Ghana using an interpretative phenomenological approach. Unpacking the social support systems. PLoS ONE 19(9): e0311123. https://doi.org/10.1371/journal.pone.0311123

Editor: Stefaan Six, Vrije Universiteit Brussel, BELGIUM

Received: March 5, 2024

Accepted: September 6, 2024

Published: September 26, 2024

Copyright: © 2024 Gyamerah et al. This is an open access article distributed under the terms of the Creative Commons Attribution License, which permits unrestricted use, distribution, and reproduction in any medium, provided the original author and source are credited.

Data Availability Statement: The authors can confirm that the data supporting the findings of the paper are available, however, due to ethical issues and respondents' anonymity, it will be made available upon request when the necessary ethical matters are followed. The informed consent forms signed by participants before they took part in the study indicated that their data will not be shared with anyone as was required by the Ethics Committee for the Humanities (ECH) at the

Abstract

Acculturation, a cultural and psychological process, can cause stress for international students studying in Ghanaian universities. This paper examined the challenges faced by these students, highlighting the many obstacles they face on campus and throughout the country, and the social support systems available to them. Using the interpretative phenomenological approach (IPA), rooted in acculturative stress concepts this paper found that international students often find life challenging and frustrating, regardless of their continent of origin. The paper revealed that university administrators and faculties do not adequately support international students, and self-efficacy is insufficient in coping with acculturative stress. Financial concerns, being unrealistically perceived as affluent, abrupt academic schedule changes, weather conditions, and frequent marriage proposals were significant sources of stress. This paper observed that international students in Ghana enjoy immediate and long-term advantages such as bilingualism, cultural awareness, intercultural understanding, high-status recognition, and easier employment in their home countries. The paper concludes that international students often struggle to adjust to Ghana's new socio-cultural and academic environment albeit there are some advantages. The findings of this study can help offices of international student affairs and student exchange organizations on university campuses in Ghana, thus, providing tailored counselling services to students in vulnerable groups.

Introduction

International students tend to have a higher chance of experiencing academic, social, and psychological difficulties [1]. Nonetheless, the connection between acculturation stress and international students attending higher learning institutions outside of their native countries is,

University of Ghana. Further information can be accessed from the ECH via email (ech@ug.edu.gh).

Funding: The author(s) received no specific funding for this work.

Competing interests: The authors have declared that no competing interests exist.

however, mediated by several social support networks [1, 2]. The psychological and cultural process of acculturation significantly increases the stress associated with adjusting to a new environment and the process of adapting to the new culture and the stressors thereof is what is known as acculturation [1, 3]. Moreover, scholars of acculturation research have argued that acculturation is a dual process of cultural and psychological alteration that takes place because of contact between two or more cultural groups and their individual members [3–5]. Acculturative stress, which is the psychological and physical discomfort experienced in a new cultural setting, can result from the pressures that international students feel because of their encounter with a new culture [6].

International students, who can effectively control their fears and uncertainties by being aware of how their hosts behave and think tend to find the acculturation process less stressful [7]. Furthermore, acculturation is also a psychological adaptive process occurring on an individual level, besides it being a cultural process. International students' level of assertiveness and sense of self-efficacy, which are part of their personality, are important intrapersonal variables that could impact the acculturation process upon their arrival into a new social and cultural environment [1, 2]. Studies have observed that international students upon arrival in their new environment experience many problems of which the most reported ones have decreased self-esteem and lack of assertiveness as well as language barriers, academic demands, homesickness, and loss of social support and status [1, 8, 9]. The main research questions that shaped the paper were as follows:

a. What constitutes acculturative stress for the international student in Ghana's socio-cultural and academic environment?

b. What social support systems influence the acculturation process of the international student?

c. What variations in support systems and coping mechanisms by international students from different cultural backgrounds?

Acculturative stress and social support: A brief survey on past literature

Extensive research studies on stress coping strategies have shown that social support is an effective stress buffer, especially for international students going through acculturation stress [1, 10, 11]. International students who possess social competence are less likely to feel hopeless, which emphasizes the necessity for educational institutions to create support systems that deal with the difficulties of cultural adaptation and improve social competence [12]. Additionally, social competence can operate as a mitigating element, assisting people in overcoming the difficulties associated with cultural adaptation and lessening the detrimental effects on their mental health. Research has demonstrated that those with greater social competence can manage the stress of cultural adaptation and are less likely to feel despair [13]. It has been shown that international pupils who receive social support from local peers experience less homesickness [14].

A vital resource for stress management and the advancement of both physical and mental wellness is social support [1, 10, 15]. It has been argued that the development of cultural knowledge and competencies necessary for adaptation depends on friendships with locals, while social support from family and fellow citizens helps international students preserve their cultural identities and customs and lessens homesickness and confusion [12, 16]. It has been demonstrated that supportive networks, such as friendships with locals, provide essential information about mainstream culture, aiding in the development of cultural knowledge and

competence for adjusting to the host society. However, several studies have revealed that undesirable occurrences like racism and signs of psychological distress are not immediately impacted by social support for international students [16, 17]. Social support helps prevent stress and maladaptive habits, which tends to benefit one's health. However, maladjustment among international students can hinder psychosocial growth and increase mental health risks, including depression [17].

Social networking sites, or SNSs, are quickly emerging as an essential tool for students studying abroad to communicate with friends and family back in their home countries to help curd acculturative stress [17]. It is important to distinguish between received and perceived social support. Social support either received or perceived protects international students against stressful life events, controls maladaptive behaviours, and provides relief through practical, emotional, and informational support, among other forms of relief, all beneficial to one health [11, 15, 18]. argued that received social support is the amount and quality of particular supportive interactions one receives while perceived social support is the receivers' assessment of the availability and sufficiency of supporting resources in their social network [11, 18].

Extensive research has demonstrated a correlation between received and perceived social support and acculturative stresses [18, 19]. Nonetheless, research has found a stronger and more stable relationship between perceived social support and outcomes related to acculturative stress [11, 18, 19]. Language competency has been argued to have a considerable impact on international students' academic, social, and psychological adjustment, with acculturation stress and social support playing moderating roles [1]. Language limitations can also have an impact on international students' interactions and socializing with other nationalities and locals [20].

Students who were more satisfied with their interpersonal social support networks reported less acculturative stress in the form of less perceived discrimination, hatred, and negative feelings caused by cultural change, according to a study done on students in large, diverse universities about the relationships between acculturative stress, interpersonal social support, and the use of online ethnic groups [21]. Similarly, it was found that among those who had used online ethnic social groups, those who reported receiving higher amounts of informational support from these online ethnic groups experienced lower levels of acculturative stress [21]. The study, however, failed to explore the possible impact that social support participants received from families and friends may have had on their lowered reported levels of acculturative stress.

The relationships between friendship networks, social connectedness, homesickness, contentment, and satisfaction were examined and the result showed that international students with a higher ratio of individuals from the host country in the social support network were more satisfied, content, and less homesick [14]. Also, participants who reported more friendship variability with host country nationals described themselves as more satisfied and more socially connected. This study establishes the critical role that social support in the form of friendship networks with host country nationals plays in helping international students deal with acculturative stress. However, generalizing the findings of this study is quite challenging because the sample size of 5% is low when Scheaffer, Mendenhall and Ott [22] argued that the sample size should be at least 10% of the population to generalize.

International students from four different colleges were studied and the results showed that social support uniquely contributed to the variance in acculturative stress among international students [8]. The study further established those students who primarily socialized with their co-nationals rather than with host nationals experienced more acculturative stress. However, others have found that social support did not have any impact on the socio-cultural adjustment of Asian international students studying in the United States (US) [23]. However, the study did not separately explore how social support from students' home country affected adjustment

from social support received from the host country. The lumping together of social support, irrespective of where it is being received, could have affected the findings of this study since there are studies that give premium to support from host nationals as effective in dealing with acculturative stress [8, 14] and those that give premium to support from home country [24, 25].

Individualism-collectivism cultural dimension has been argued to have some implications for international students' acculturation experience and its concomitant stresses [24]. Research on international students' acculturative stress suggests that students who originate from societies with collectivistic values, such as countries in Africa and Asia, experience difficulties when in contact with societies that emphasize individualist values such as countries in North America and Europe [2, 26]. Moreover, international students from Asian and African countries studying in Germany experienced higher acculturative stress compared to their counterparts from other European countries [27].

International students from collectivist societies studying outside their home country reported that competition in foreign universities and educational settings could sometimes take away the opportunity to learn and to relate, thus experiencing acculturative stress [28]. Moreover, international students from individualist societies that value self-promotion, self-assertion, and competition studying in societies that are collectively inclined who place value on modesty, interdependence, and in-group harmony are likely to report acculturative stress.

Religiosity in the form of church attendance, involvement in church-related activities, and frequency of prayer and religion have been observed by [29] as the use of cognitive and behavioural techniques when confronted with stressful life events, are important predictors of acculturative stress. Research done among African Americans suggests that the use of religious resources such as prayer and religious service attendance tends to temper the effects of discrimination as well as other forms of psychological stress [30, 31]. Conversely, it has been argued that religious involvement appeared to exacerbate the effects of acculturative stress [32].

It has been established that international students studying in China who were part of an organized religion, especially Christians and Muslims experienced more difficulties finding partners and locations for religious activities leading to acculturative stress as they scored high on homesickness [33]. The same study found that persons who identified as Hindu or Buddhist scored higher on cultural competence. The literature on religion and acculturative stress is quite conflicting. Thus, it may be interesting to further explore religion and acculturative stress among international students in Ghana where about 95% identified as religious.

Theoretical framework: The acculturative stress model

Scholars proposed the acculturative stress model to understand the processes and outcomes of acculturation [34]. This model emphasizes the importance of examining acculturative stress as a manifestation of acculturation when individuals or groups encounter another cultural group. Acculturation involves cultural and psychological changes, as individuals' values, norms, beliefs, and traditions may differ from another culture. Acculturation can improve life chances and mental health, but it can also cause acculturative stress, leading to anxiety, depression, feelings of marginality, alienation, heightened psychosomatic symptoms, and identity confusion [34]. The model (Fig 1) illustrated by what [34] refers to as acculturation experience, on the left-hand side of the figure, shows that acculturation occurs in a particular situation or cultural environment.

Acculturation experiences vary among individuals, ranging from minimal to significant changes in their new environment, whether they are part of a migrant community, native settlement, sojourners, or international students. In the middle section of Fig 1 referred to as stressors, the model illustrates that an individual could experience stressors which could come

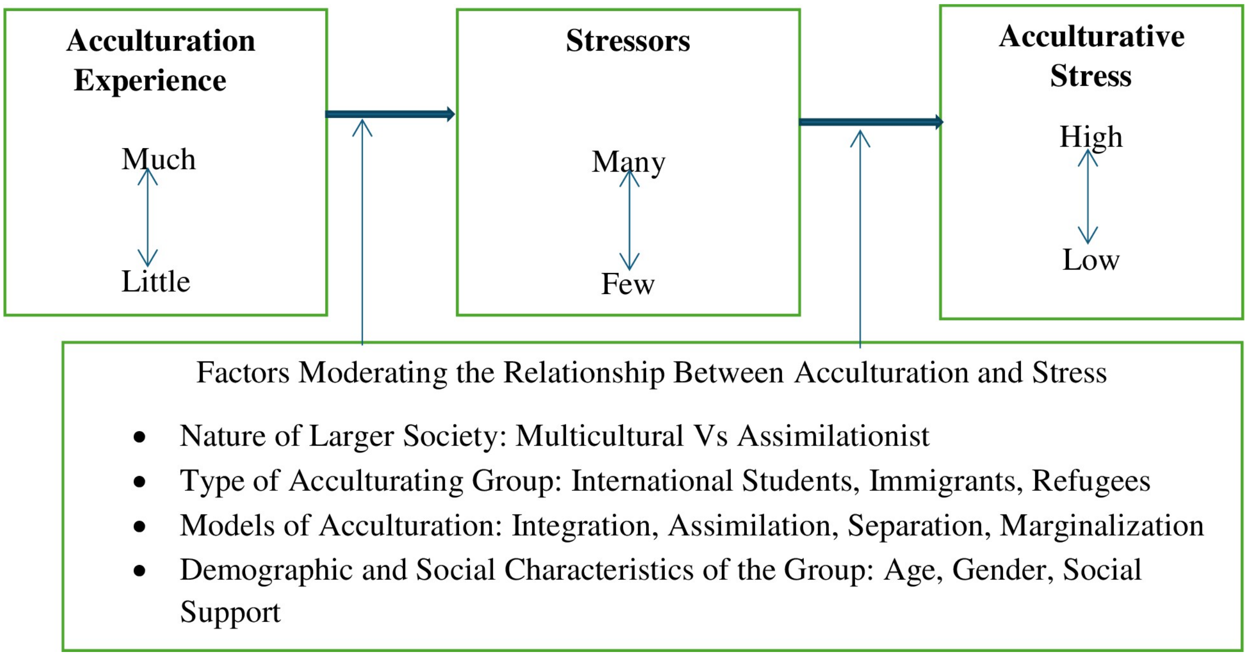

Fig 1. Acculturative stress model. Source: [34].

https://doi.org/10.1371/journal.pone.0311123.g001

about because of the varying degrees of acculturation experiences, they may have had. However, acculturative changes can be perceived as stressors for some individuals, while others see them as opportunities for personal growth or benign stressors in their new environment. On the right side of Fig 1, the model shows the variations of acculturative stress, high or low, that may become manifest due to acculturation experience and stressors.

This model is logical but not a "quid pro quo" phenomenon. It has been argued that the model is probabilistic, as the relationships between acculturation experience, stressors, and acculturative stress are influenced by various moderating factors, including the larger society, group type, mode of acculturation, demographics, and individual psychological characteristics [34]. The moderating factor for a host society's tolerance for diversity is whether it embraces pluralism operates on a multiculturalist ideology, or if it pressures conformity to a single cultural standard.

It has been suggested that individuals experiencing acculturation in culturally diverse societies may experience lower stress levels compared to those in societies with forced inclusion. The acculturating group, those who consciously choose to undergo acculturation, are less likely to experience stress compared to those with limited choice [35]. Thus, Berry et al., [34] assert that the variations in the degree of voluntariness, movement, and permanence of contact tend to affect acculturative stress levels of acculturating groups. Furthermore, if the group is sojourners or international students, such persons could only be in temporary contact with the host culture and less likely to have permanence of social support and therefore could experience more mental health challenges compared to those who are permanently settled [34].

The strategies adopted by individuals or groups during acculturation can moderate the process. Berry et al. and Sam and Berry suggest that cultural dimensions, such as maintaining heritage cultures and interacting with host cultures, influence acculturation strategies, leading to four different modes of acculturation [34, 36]. The literature identified four strategies for acculturation: integration, assimilation, marginalization, and separation, which involve

maintaining contact with one's original culture while actively participating in the host culture [34]. The integration model of acculturation involves individuals maintaining contact with their original culture while actively participating in the host culture.

Assimilation is a strategy where individuals seek close interactions with host culture members without preserving their cultural identity, also known as "going native" in anthropological terms. Individuals who prioritize their original culture and avoid interaction with new society members adopt the separation strategy [36]. Marginalization refers to a lack of interest in cultural maintenance due to cultural loss, and in maintaining relationships due to exclusion and discrimination [36]. Individuals with a marginalization or separation inclination are more likely to experience stress and adjustment difficulties compared to those who are integration and assimilation inclined [34].

The fourth factor moderating the effects of acculturation and its attendant stresses is the demographic and social characteristics of the acculturating individual such as age, gender, level of education, and intercultural experiences before entering the host culture among others. Berry et al., [34] posit that these characteristics are likely to moderate the acculturation process and outcomes. Finally, an individual's psychological characteristics such as attitudes towards change whether negative or positive; cognitive appraisal of the situation whether controllable or threatening; and coping skills of the individual impact how a person adapts to life in a new culture as depicted in Fig 1. Individuals' responses to cultural changes vary greatly, with some recognizing changes as easy, others as a challenge, and some experiencing psychopathology like clinical depression or anxiety due to their varying perceptions.

Design and methods

Positionality statement

There are four (4) authors for the current study, and we hold PhDs in various fields of expertise (e.g., Adult Education and Human Resource Studies, Psychology, and Public Administration). The lead author has over two decades of experience working with international students who come to Ghana to study under the sponsorship of the CIEE, the largest not-for-profit international educational exchange organisation in the world. Two of the authors have been international students themselves. One of the two pursued a PhD in Norway and the other pursued their Masters and PhD in South Africa. The fourth author also has extensive teaching experience at the University of Ghana where her classes have always had international students. More specifically, for the past 8 years, she has been teaching an internship course for only study abroad students who come to study on the CIEE program at the University of Ghana. Our collective lived experiences as administrators, researchers, academics, and international students, guided the selection of this topic since we acknowledge the important role social support plays in dealing with acculturative stress among international students as they transition from one culture to another. This in-depth personal understanding of the lived experiences of international students informed the construction of the interview guide used to collect data for this study, the interviewing technique adopted, the theoretical approach used, and our choice of data analysis. We recognised that our positionality influenced not just how we regarded the research participants, but also how they perceived and interacted with us. However, we developed an inclusive and courteous study setting, remaining open to all participants' viewpoints and experiences. At the same time, we made efforts to bracket our existing biases and had regular open discussions to ensure that we presented findings that accurately represented the lived experiences of our study participants.

Design

The qualitative data analysis grounded in an interpretative phenomenological approach (IPA) was utilized in this analysis [37]. Since IPA is based on phenomenology, it was adopted to gain insight into the lived realities of international students as they navigate the pressures associated with acculturation. The methodology made it easier for the researchers to comprehend how foreign students on the two university campuses in Ghana interpret their acculturative life experiences both on and off campus. The theory of phenomenology guided data collection as participants were made to respond to questions such as: "What is your experience of being an international student studying in Ghana?". Ethical assurance on the consent forms they had read, understood, and signed also allowed for the open and free expression of experiences. Responses provided were probed rigorously to learn more about their experiences and how they dealt with stressful intercultural situations academically and with the larger Ghanaian socio-cultural environment. The analysis presented in this paper was part of a doctoral research project titled "Dealing with Acculturative Stress Among International Students in Ghana: Influences of Assertiveness, Self-Efficacy and Social Support" that was carried out at two universities in the Greater Accra Region of Ghana between October 3, 2016, and January 20, 2017 (see Table 1).

Sampling procedures and participants

Through a convenient sampling approach [38], a sample was drawn from the international student population in two universities (one private, one public). A total of fifteen (15) participants, 8 males and 7 females, were interviewed. Two (2) of the in-depth interviews, out of the total, were conducted among international students at Wisconsin International University College (WIUC) (1 African male and 1 African female). The rest of the in-depth interviews were all conducted at the University of Ghana (UG). Table 1 shows the profile of participants indicating their gender and continent of origin. Interviews were conducted with seven (7) international students from Africa, three (3) Americans, three (3) Asians, and two (2) Europeans.

A convenient sampling approach grounded in snowballing technique was employed to solicit information from the 15 study participants [39]. The selection of participants was done through convenient sampling with the help of a Research Assistant (RA). Prospective participants were recruited and informed of the study and asked if they would be interested in participating. Suitable days and times were scheduled with students who agreed to participate in the study. This procedure was however successful with only four (4) participants as there were situations where participants who had initially agreed could not make it due to class work, lecture attendance, or personal reasons. Thus, a snowball sampling technique was then employed by requesting that these 4 participants tell their friends about the study and ask if they would like to participate. Eleven (11) of such participants were recruited for the interview. All the participants had been studying at the two universities for a period of between 3 months and 4 years.

Table 1. Profile of international students interviewed (N = 15).

Gender	African	American	Asian	European	Total
Male	4	1	2	1	**8**
Female	3	2	1	1	**7**
	**7**	**3**	**3**	**2**	**15**

Source: Field data, 2016–2017.

https://doi.org/10.1371/journal.pone.0311123.t001

They ranged in age from 19 years to 35 years. Two (2) of the African participants (1 male and 1 female) were graduate students with the male pursuing a master's programme in Agricultural Science and the female pursuing a PhD in Sociology. The remaining were undergrads who were in Business School, Medical School, Psychology, and Sociology majors while the visiting international students were taking courses mainly in the Colleges of Humanities and Basic and Applied Sciences.

Data collection tool

In-depth interview. A self-developed in-depth interview (IDI) guide was employed [40]. The IDI guide was used to ask participants to talk about what it means to be an international student; whether there are any benefits; whether there are any acculturative stress challenges they face as international students, what these challenges are; and finally, how they are dealing with these challenges.

Semi-structured interviews. To complement the in-depth interviews, semi-structured interviews were conducted with participants [41]. Deep engagements were established with the participants during the interviews through a very strong rapport that was built at the very beginning of the interview session.

Documentary evidence. To augment the fieldwork interviews, the study drew on available documentary data pertinent to international students and acculturative stress in Ghana. These relevant secondary data including reports, policy briefs, publications, newspaper articles, and unpublished research reports were also examined using a documentary analysis technique through thematic analysis [42].

Transferability

The analysis approach employed numerous qualitative techniques, including member verification. The study themes and categories developed in the transcripts were extensively examined utilizing formal or informal validation procedures [43, 44]. Once more, because of the numerous unique data points used, the researchers employed a data triangulation technique. Furthermore, participants engaged in the fieldwork for an extended period, which improved the rapport between the researchers and respondents and enabled the collection of detailed data [44]. Additionally, peer debriefing in which colleagues with various research interests interact with the data was employed. This assisted the researchers in identifying potential biases and providing clarification on how to interpret the data [44].

Data analyses

The study employed a qualitative thematic analysis approach grounded in IPA [45]. The field interviews were audio tape-recorded and transcribed verbatim to ensure that participants' responses were captured in their true and original state. Strong rapport allowed participants to narrate their experiences freely and openly. The shortest interview lasted 40 minutes while the longest lasted an hour and 28 minutes. Before each interview began, participants were given consent forms to read and understand, ask questions if they needed clarity on anything, sign two copies of the consent, keep one, and return one before the interview began. The participants were assured of the confidentiality of the information they provided, the anonymous nature of the interview, and the fact that they could discontinue the study even if they had started the interview.

The field data were later transcribed, and the transcripts were named using the participant's gender, continent of origin, and a number depending on the order in which they were interviewed. The first male participant from the African continent was named "M1, African", and

the first female participant from Asia was named "F1, Asian" and so on using IPA analysis strategies [37]. The steps followed as outlined by the [37] the guide was that each transcript was read and re-read to assess the key emergent themes for the whole group allowing for what [46] describe as an iterative process as there was a very close interaction between reader and text. Secondly, linkages between quotes that shed light on group-level themes were established and they were further linked to recurrent themes. Finally, themes were verified and summarized, and analytical connections were made among the themes [37], to ensure a sound, coherent, and analytical write-up.

Using [45] thematic analysis, the codes derived from the data were categorized into themes. The coding process began with a three-step reading of each transcript and matching each transcript with the field notes. This gave the data's hand coding a strong foundation. The study's objectives were achieved by sorting and categorizing these codes based on themes. After a theme analysis approach was used to examine the codes taken out of the transcripts, they were grouped into more focused subjects for further analysis. The first step in this research technique is to compare codes with arguments to find similar patterns. The different acculturative stressors and their suggested remedies were identified through a review of the numerous themes derived from the field transcripts. As a result, three (3) main themes, twelve (12) sub-themes, and eighteen (18) sub-sub-themes were derived from the in-depth interviews conducted (see Figs 2 and 3) after continuous comparisons with the various data sets were used to build the themes. This paper ascertains how themes link to the underlying ideas by employing them as a guide.

Thus, employing the thematic analysis approach this paper identified intriguing patterns in data, thereby guiding researchers to address the key objectives of the study. Thematic analysis provided the researchers with an organized, yet flexible, approach to interact deeply with the data and modify the approach to fit the objective of the data analysis [45]. The analysis of the in-depth interviews took the sequence as delineated by Berg [47] (p. 240):

- Collation of field research notes and transcription of audio interviews;
- Developing data codes (logically inductive) from the field data;
- Transformation of data codes into comprehensive labels or themes;
- Organisation of themes, labels, and categories by identifying and sorting similar phrases, patterns, relationships, and commonalties or disparities;
- The fragmented categories and labels that were sorted and scrutinised into meaningful and manageable transcripts to segment patterns, thoughts, and processes; and
- The segmented patterns identified in the transcripts were carefully interpreted juxtaposing them to previous studies, theories, and frameworks to construct some level of generalisations.

Using a triangulation method [48] the interviews conducted with all the study participants were triangulated with the documentary literature to ensure data accuracy and robustness of the interpretation [49, 50]. To ensure participants' confidentiality, this paper utilised pseudo-variables to present the empirical findings of this paper. To evaluate the study's robustness, the "four-dimension criteria" of qualitative research; credibility, dependability, confirmability, and transferability were employed [51]. The study established data saturation, ensured a high level of consistency, and achieved intercoder agreement among different coders. Moreover, other modified strategies used included well-organized data collection procedures, defined participants, and participation as well as substantial data reliability through multiple coding of the transcripts [43, 44, 52].

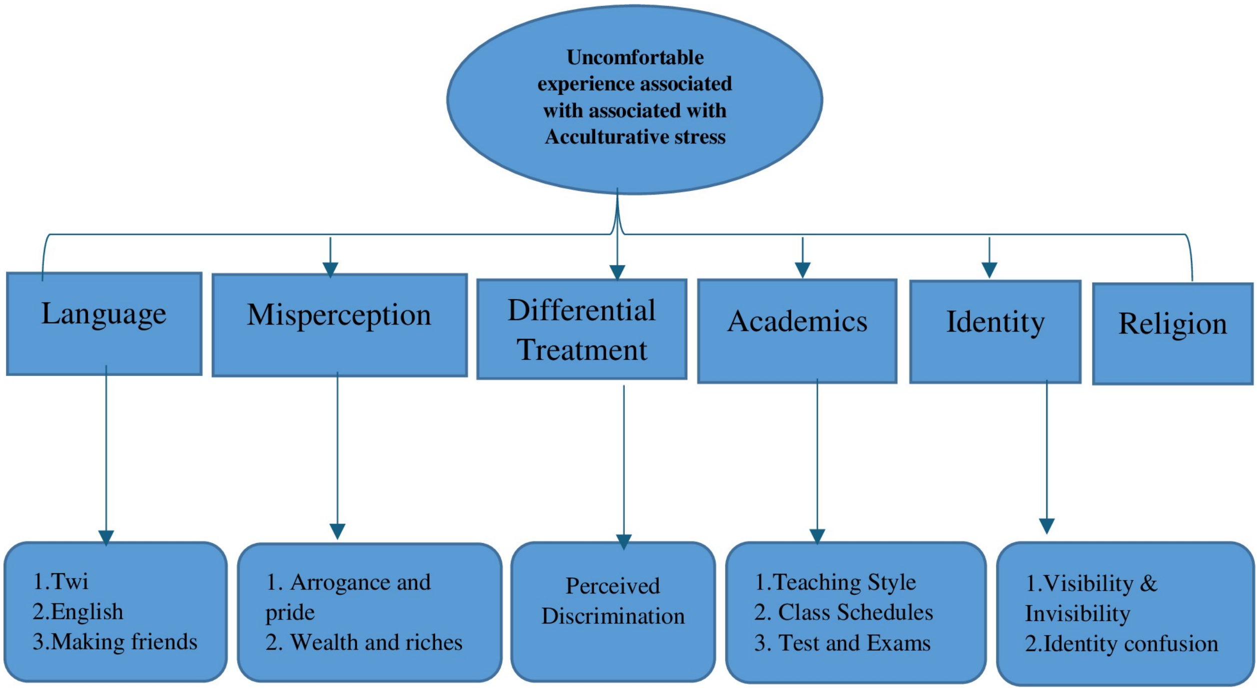

Fig 2. Theme and sub-themes of uncomfortable experiences associated with acculturative stress. Source: Field study.

https://doi.org/10.1371/journal.pone.0311123.g002

Ethical considerations

Ethical clearance to conduct this study was obtained from the University of Ghana (UG) Ethics Committee for the Humanities (ECH), Legon, Accra Ghana (Ethic reference number: ECH O59/15-16).

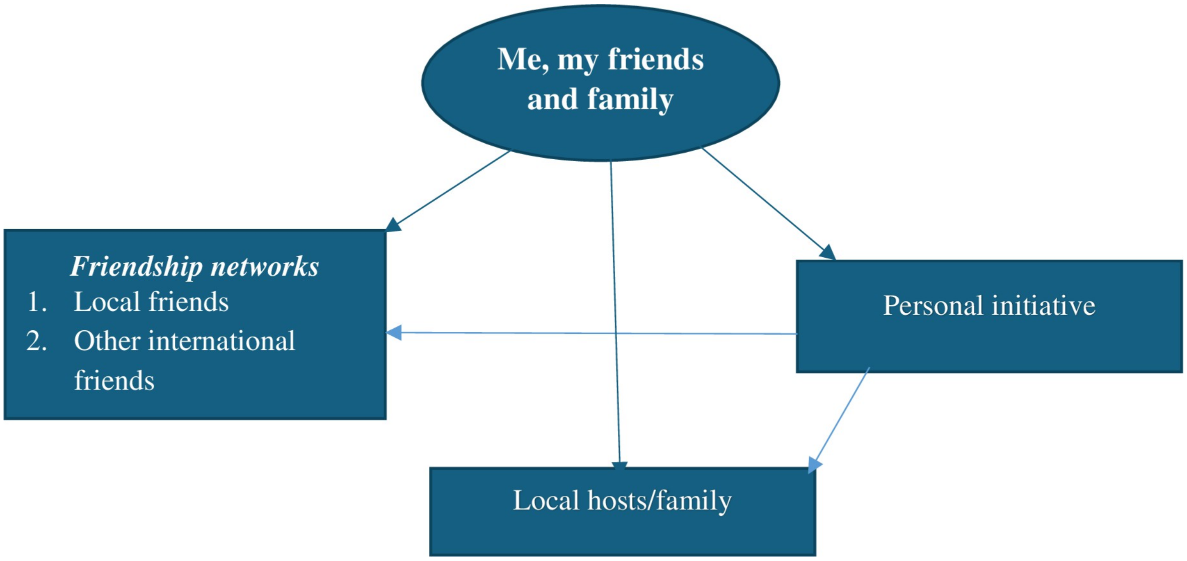

Fig 3. Themes and sub-sub-themes of social support.

https://doi.org/10.1371/journal.pone.0311123.g003

Findings

Uncomfortable experiences of international students living and studying in Ghana. International students irrespective of their continent of origin found living and studying in Ghana a challenging and frustrating one, describing it as an uncomfortable experience. Financial considerations were a major source of stress for most international students due to being wrongfully perceived as affluent and therefore easy target of exorbitant pricing for basic items when they know fellow local students will not be asked to be that much. A quote below illustrates this sentiment.

> *". . . And there's also that thing when you go to the market. When the woman finds out that you are not a Ghanaian, they'll triple the price for you. It's the same thing with the taxi drivers too" (F3, African).*

International students in Ghana feel they are not receiving proper appreciation for their financial contributions to society and universities, believing they should receive preferential treatment, unlike their Ghanaian colleagues. The frustration of an African student is expressed in the quote that follows.

> *Well, honestly, to me I can say I'm having a very terrible experience. . ..it is believed that Nigerians pay most of the money as international students here because when you think about it, ... but then Nigerians are the ones getting the most insults from the local people around (M2, African).*

Another reason why international student describes their experience as uncomfortable is academically related as seen in the quote below:

> *I feel kind of outside of the loop sometimes. I know that I've had classes cancelled or moved and somehow all the local students knew, but none of the international students did. . . I feel it's a little bit harder to get information or I don't know much about how this system works (F5, American).*

For many American and European students, false notions of affluence were a key cause of acculturative stress:

> *I think there is always the issue of paying for certain course material and I guess some students. . .when the subject of money comes up, a lot of students protest a little bit. They don't want to pay for it, but I think that people kind of assume that it is nothing for me (M8, European).*

Ghanaian men's daily marriage offers significantly exacerbated acculturative stress among female international students, especially those from Europe and America. An American student expressed their frustrations in the interview extract below.

> *It has been a frustrating period for me. . . Since I've been here, I've never gone a week without being propositioned for marriage (F6, American).*

Some international Asian students in Ghana experienced difficulties due to the hot weather and slow internet speed on university campuses:

When I came first it was embarrassing because of the weather. The weather is so hot, very hot and it's difficult to walk around here (M7, Asian).

In Korea, you can use the internet or Wi-Fi. It's so fast. But the international hostel and International Programme Office [IPO], we don't have fast Wi-Fi and internet speed so it's a little bit uncomfortable (M4, Asian).

There were other stresses including language difficulties, misperceptions, differential treatment, academic challenges, identity, and religion, that were derived and discussed below as a result of probing further the broad theme of 'uncomfortable experiences.

Language difficulties. International students in Ghana experience acculturative stress due to their inability to speak the local language (Twi), leading to uncomfortable interactions with taxicab drivers, market women, and traders, and increased costs for services:

Yes. The language. Sometimes we go to the market, and we will be speaking English and some of them don't understand what we are saying. . . . so, for me, I think language is one of the toughest challenges for now (M1, African).

Relatedly, some international students, particularly those from French-speaking African countries and Asian countries struggled to communicate in English leading to social isolation and limited friendships despite the presence of local and international students on university campuses. Below are two interview extracts from two African students with language difficulties:

As an international student coming from a French country, I have difficulties to be sociable because the language is like. . .because my English is not good, I might fail to speak to other people like Nigerians, Ghanaians or. . .so I am always in my room with my French people (F2, African).

. . . and also, it's very difficult to get real friends. Yeah. Like Ghanaian real friends. They're just there to you know, I need this, and I need that. But real friends to be there when you need them and, you'll be there for them too. The two ways (F3, African).

Misperceptions. Another source of acculturative stress for several of the international students was the wrong perceptions that they reported Ghanaians, non-students, and fellow university students, had of them as being arrogant and proud. Arrogance and pride are such negative stereotypes that being labelled as such by locals makes them have uncomfortable experiences as it does not present a true reflection of them. The quotes below depict the experiences of the students:

Some people have this perception of Nigerians that we are arrogant, we're proud so sometimes some people meet you with that stereotype before they get to know you and it's always a shock for me (F1, African).

Participants also reported that the perception of a foreign person signifying wealth and/or riches was a stressful experience for them as it was a wrongful assumption. This lived experience is illustrated by quotes by an African and American student.

Most people have the perception that Nigerians are very rich people so the strangers I meet are like 'Oh you, you have money (F1, African).

The most common one is about me being from America and that I have a lot of money at my disposal, which isn't true. . .. I don't come from a wealthy family so it just never occurred to me that people might think that (F5, American).

Differential treatment. International students have also reported receiving different treatment in which individuals, even academic faculties and officials at the university, look out for them, provide them with unsolicited information, and pursue their welfare and interests, whereas local students do not receive the same care. Such differential treatment, even if favourable, causes them some distress, as evidenced by this finding:

One time a professor called me before the class, and he asked me how the class was going. And that was a little bit strange because I knew he would never ask that question to other Ghanaian students (M7, Asian).

Some of the local students always think the university is being. . .how should I put it? They would say that oh, just because we are whites from foreign countries, the university is praising us like we are gods (F7, European).

Within the international student body, there was the perception of discrimination where some of the African international students stated that the university staff and certain offices that serve the needs of international students gave the "white" international students special attention compared to them.

They treat the white [students] like they are more international than us. All of us are international students but once the whites come, they would want to give them more special treatment than us. For example, during registration. . .they told us that we should go outside and that they were going to call us one by one but when the white [students] came they just allowed them to enter and gave them a lot of special treatment (M1, African).

Such incidences and alleged cases of discrimination against African international students by Ghanaians whom they perceive to be their fellow Africans were significant causes of stress that made their experience unsatisfactory and unpleasant. They began to wonder about the foundation of Ghanaian society and why certain foreigners were treated better than others. Other international students who have observed such treatment by Ghanaians, in the larger society towards other African international students think it is unfair that African international students receive such treatment from their fellow African hosts and felt quite distressed about it. The quote below drives the point home:

I think once you are in the international hostel you are getting the same treatment on a hall basis. But when you are out of the university setting, I think we who have a clear physical appearance are treated differently than those Africans who are not Ghanaians. . . (F4, Asian).

Academic challenges. Since international students are first and foremost students whose success in academics was very important to their very decision to study in Ghana, academic issues were also found to be a major source of stress for a good number of the participants interviewed. Issues such as lecturer and student absenteeism especially on the first day of class, changes of class meeting venues, and the absence of an online portal where all courses offered

at all departments could be found were reported as stressful for international students, especially those from the Asian, European and American continents. The stressful academic experiences were the result of students comparing their previous universities to the universities sampled in this study. This is what some participants said:

> *When I was registering for courses, I had to go to each department which was hard for me because I didn't know where the department was; or where should I go so. . .I wondered why I had to go to the department personally. We first register by website. That's all. In Korea, it's just registered by website. But I didn't know why I will go to the department. So, I wonder a lot (M4, Asian).*

> *First and foremost, UG [University of Ghana] is rather unorganized, at least compared to my school in the United States. For instance, course schedules can only be found on the bulletin boards outside each department as there is no master list online. For me, this was frustrating (F5, American).*

The lack of class exercises in the form of continuous assessments which will enable students to know how well or badly they are performing in a course, except one (1) Interim Assessment (IA) constituting 30 to 40 percent final of grade and the final examination constituting 60 to 70 percent was a source of academic stress for some of the respondents as expressed in the interview extract below.

> *Personally, . . . I would like to have homework that would get me to study a little bit more. It gives me an incentive to study and it's something I don't get here. The homework is scarce in my school. You get homework let's say for one or two courses during the semester. Also, the 70% final is very intimidating (M8, European).*

The different teaching style where in some cases notes were dictated verbatim was a major source of challenge for some of the students. These were stated this way:

> *Sometimes it's really hard to get the specific words that professors say. . .. That is actually one of my big challenges (M7, Asian).*

> *The style of teaching has really been different too. She has prepared this course book for us; and in every single class, she just kind of picks a few students to take turns. They just read out loud from the course book. . .for 10 weeks. It was not what I was used to, I am more used to more engaging and interactive (F5, American).*

Not having a good understanding of the English language, which is the medium of instruction in Ghana, made the academic experience for some of the international students a difficult and stressful one.

> *In class. At the beginning, the lecture was so difficult sometimes. . .Your first time to take a lecture in English. . . You can get only one word. . .you can miss the whole tense because you don't understand the words. It's difficult for us (M3, African).*

> *But I think the vocabulary I don't know so much. In the class, I don't understand what the meaning of that is. Sometimes people say things that I don't know. Vocabulary is my problem (M7, Asian).*

Closely related is the fear of getting poor grades as their English language comprehension is, according to some of them, not up to par thereby making it difficult for them when answering essay-styled questions during final exams. This fear is illustrated by the quote "During the exam, sometimes especially in the essay parts, it is difficult" (M3, African). Another also states:

I always forget how to spell the words and the sentences; what I remember before the exam. It's like I'm nervous so I forget. So, it's not easy to write. I think if it's an English student, you just read it and they can memorize it; so, it's easy to write. But for me I remember, and I repeat again and again before I can write. So, the English is my problem (M7, Asian).

Identity (visible or hidden). A person's sense of self-worth is directly influenced by their identity, which includes who they are, how others see them, their unique or common personal traits, as well as the values and ideas they hold. A person's identity may be influenced by being in a different culture and may even have an identity crisis. The physical characteristics of being a white person and being called names by locals made some of the European, American, and Asian international students feel disrespected leading to an uncomfortable and challenging experience for them. Such feelings are represented in these quotes,

I don't know but everywhere I go I am seen since I am tall, blonde, white woman from America and people call me obroni. . .obroni (white) (F6, American)

I am very noticeable and stand out not because of my academic achievement or skill, but just because I am a foreigner (M8, European).

Another student who feels disrespected by name-calling also expressed that in the interview extract below.

I think some Ghanaian men are not polite, they always call me China or Chinese. . .. China, China like that. They don't call me by my name" (M7, Asian).

For some of them, being in Ghana led to some aspects of their identity becoming hidden and/or people confusing their nationality with that of other nationalities. Since identity is such an important marker to all persons, a loss of some or all of it for any reason, tends to engender stressful experience. For example, according to some of the students:

It's a little bit similar to what I said but, on the streets, apart from people calling me white, people frequently call me China or China man and that is also a challenge for me because I'm Japanese. . . But I found myself feeling uncomfortable when people just called me Chinese or China (M5, Asian).

It's really new for me that people call me a white person. Because I have never identified myself as a white person (F4, Asian).

Such new identity references have pushed some of the students to frontiers they have never averted their minds to, especially on the issue of race and racial dynamics which was a source of confusion for them.

. . .here many people have called me the white person and made me change my perspective or my feeling towards so many issues or how I see the world. And for example, the racial issue;

> *black, white dynamics. . .I have never felt because I'm not part of them. I'm not white, I'm not black. But here, maybe people don't really have that deep connotation about them calling me white but for me, if they call me white, that has a totally different meaning on me (M5, Asian).*

Religion. Religion, especially Christianity played a major role in causing stress for a good number of the international students as the issue of religion came up in their everyday interactions and even in classrooms. Some of the participants were frustrated because the time that should have been spent teaching them was spent on religion and proselytization to the extent of even being made to feel unworthy. After all, they were not ascribed to a particular religion. The quote below illustrates the frustration of a study participant:

> *I have a Teaching Assistant for my history course, he spends a lot of the tutorial sessions talking about religion. . . he somehow incorporates it into his discussion, and sometimes when he is using examples he'll say like if you want to be really successful in your community, you become a high-ranking member of your church and help spread the word of God. . .you know stuff like that. And he said to the class that if you are not religious, then your life isn't worth living. Why am I paying to listen to someone take time out of my day to listen to him tell me that my life is not worth living (F5, American)?*

International students with different religious orientations in Ghana face stressful acculturative experiences, feeling perceived as non-believers due to their different religious orientations. This found expression in the following statement:

> *Ok, church. So many people ask me to go to church with me but I'm not very like that. In Ghana religion is. . . But in China, religion is not important. . . in our university, we don't have this course, and we have not only Christians. We have Buddhists. And I'm not a Christian but people always ask me do I know Bible. What's my religion? If I say Buddhist, they told me I should go to church. I say I'm Buddhist, but they always ask me to go to church with them; and that I should believe in God (M7, Asian).*

The frequency of being invited to Church and to other Christian programmes and activities as well as sometimes being bluntly told that the devil is controlling non-Christians were also some uncomfortable experiential moments for several of the international students. The quotes below illustrate the frustrations and challenges of the students:

> *The church part, they take it more seriously here than we take it in Benin. . .. Here when you don't go to church on Sunday, they'll say you have an evil. . .(F3, African).*

> *At one point I was having dinner and my host mum's daughter was sitting with me and just telling me about Christianity, and she said that people who don't accept Jesus Christ were still being controlled by the devil, and they had no control over their lives. . . that really shocked me and I was angry a little bit that she said that to me (F5, American).*

Social support in dealing with acculturative stress

Finding out how the students were coping with the socio-cultural and academic difficulties that made life difficult for them was essential because being an international student in Ghana seems to be a challenging one judging from the earlier discussions. The main strategy adopted by respondents to cope with the stressful circumstances they were having was to seek social support from friends, particularly local friends, and fellow foreign students as well as from local hosts or families.

Local friendship support. Making local friends proved to be the primary social support channel international students used in dealing with stressful academic and acculturative experiences, even though it was initially difficult. To learn about classes, navigate the academic environment, and grasp what is going on in order to perform well on quizzes, assignments, and exams, developing local friendship networks was helpful. The responses below are what some of the students gave:

I tried making at least one friend in each class and studying together with them so I could fill out what I didn't get. . . At the beginning, it was not successful at all but towards the end I made several friends in each class and actually we are studying together for the final exam (M4, Asian).

A colleague Asian student further elaborated on the position of M4 in the interview extract below:

. . . And I have a lot of friends at level 200, so I always talk with them. They give me advice. In the first, I should look for a course advisor in business, my programme. So, they told me how to do it. And they also told me that after class, if I have any problem, I should go to see the lecturer that teach us and talk with them . . .This semester yes. This semester, after class, I read books and if I have a problem, I go to see them. And I find a good way. . . I think it's a good way for me. I should do the past question, write my answer and when I'm not sure I should go and see my lecturer and show him my exercise, so they check the answer for me. Maybe I will do it next semester (M7, Asian).

This is further emphasized by an international student from Africa:

I try to make friends because if you don't make friends, let's say in school, you won't get information; you won't know what is going on. So, I have to (F2, African).

Local friendship networks proved to be equally beneficial for the students in helping them deal with local language difficulties and day-to-day problems. The quotes that follow highlight the value of social relationships as expressed by M4, M2 and F3 respectively:

When I came first, some local friends helped me to get to Accra mall and how to get to use "trotro"; and the market. He was ready to teach to me; so, he taught me (M4, Asian student).

I have made a lot of friends, so I used to take some of them. I tell them that please help me to interpret this, help me to press this down, help me to do this and they will do it; that's all (M2, African).

Most of the time when I want to go to the market, I go with a Ghanaian friend. So, she will discuss the price with them. So, when I get to know the price, the next time I can go alone (F3, African).

Fellow international friendships support. Friendships with fellow international students, and especially those from the same countries who have been in the country and at the university for a while provided a more practical avenue to deal with the everyday challenges experienced. As is expressed in these quotes,

> "Whenever I really need someone to talk to, I would probably go to one of my friends on the programme" (M8, European), and "For most of the issues I mostly go to friends on the programme" (F6, American).

Some of the students preferred turning to their fellow international students when they needed help with one thing or another. The quote below further buttresses the reliance of international students on their fellow international students for assistance.

> *More practical advice. . . I think an older student. . .the older students really helped me a lot; older Nigerian students who I met. They really helped me a lot in terms of getting to know stuff, getting to know how things work, getting to know the places to not go to, getting to know the places to go to. If you want to find this or go out to have a drink or go have dinner. . .you know familiarizing yourself with the places or things that you need to absolutely be familiar with (M1, African).*

Local hosts and local family support. Besides friendships with local and fellow international students, participants reported that local hosts and local family networks they have in Ghana helped in dealing with some major as well as everyday challenges. The quote below is indicative of the important role local hosts/families play in helping them handle some local issues.

> *Sometimes I had to call him that reverend I need to buy this, I need to do this, you know. Sometimes he'll come and pick me up; we'll go there. I remember the time I went to buy a lot of things for my room. He picked me up; we went to Makola and then we bought stuff. Yeah, and they also took me to the place where I had to get my permit. . .my non-citizen card. So, he took me to that place, and I did that. So, he was a bit more helpful with moving around. He told me how much I should averagely pay for things. He told me oh, a cab to this place shouldn't be more than this. . .or alternatively, you can take a "trotro" although you are not used to a "trotro" (M3, African).*

An American student attested to the Ghanaian host family's sociocultural approach to handling major acculturation issues in the interview extract below:

> *. . .If it was something major, I'm sure I would probably go to. . .. I would go to my host mum first (F6, American).*

Personal initiatives. Participants reported that taking personal initiative to read, watch English-language movies with English subtitles, take local language classes, and deliberately try to make friends helped them cope with some of the socio-cultural and academic stresses they encountered. The following quotes highlight the participants' efforts:

For the local language, I had a great interest in learning Twi but none of my friends were ready to teach me. So, I did UGRC Twi in level 200 but after the examination, I don't seem to remember them anymore (F4, Asian student).

A student from Africa reaffirmed a similar endeavour to lessen some sociocultural pressures:

Yeah, in the beginning, it affected the grades but now, to deal with that we read a lot. We google, find a course, and read before going to class. So that if you go, when the teacher is speaking, you can understand what he means. And I like to watch movies in English a lot; the subtitles in English. . .I have a dictionary, and when I don't understand some word, I read it. I think that helps me (M3 African student).

International students' initiatives significantly influenced their friendship networks with Ghanaian students, non-students, and fellow international students, which helped them cope with acculturative stress. Similarly, personal initiative was influential in the international student seeking support from host family when encountering a difficult cultural situation. Fig 2 summarizes the themes and sub-themes of this finding.

Discussion

International students are typically a susceptible group since they have a difficult time adjusting to Ghana's new socio-cultural and academic environment, albeit there are certain advantages. The African international students experience high levels of acculturative stress because they feel discriminated against by members of the local Ghanaian community, local students, and some university officials, in contrast to other international students, especially white students, who pay equally higher tuition fees as paid by every international student. Since they all pay the same tuition, all international students ought to be treated equally and given the same advantages. In a similar study, [1] found that international students face higher academic, social, and psychological challenges due to varying socio-cultural dynamics.

The locals may have assumed that Ghana would be more familiar to Africans and that they would not need any assistance navigating and understanding the Ghanaian culture, which the African students perceived as unfair and unequal. This is because Ghana and other countries have many cultural differences and complexities in terms of food, language, marriage, religion, and other traditional practices. Ghana's religious landscape makes it culturally distant from most societies where international students come from. In this study, religion and religious activities (e.g., church invitations, preaching at various places, and proselytization activities) caused stress for international students as they were perceived as unwanted, and invasive and made them feel unworthy.

In Ghana, 95% of the people ascribe to one religion or the other [53] and tend to wear their religion on their sleeves in the ways they express their religiosity. Meanwhile, in most of the societies where international students come from religion and religious expressions tend to be private, and persons who do not associate with any religion and/or identify as atheists, free spirits among other such religious orientations are not made to feel unworthy. Our finding is consistent with [54, 55] finding suggesting that the greater the difference between home and host cultures the greater the stress. Therefore, it is not surprising that religion, a central feature of the Ghanaian socio-cultural landscape, was a source of stress for international students because of how Ghanaian people express their religion when interacting with international students. Similarly, for the female international student's frequent marriage proposals were unwanted and

invasive to their privacy and this added to their feelings of acculturative stress. The finding is consistent with a study where the authors argued that there is a high possibility of acculturative pressure on international students when encountering a new cultural setting [23].

Moreover, the expectations on the part of African international students about Ghanaian societies and academic cultures to be the same or like what they already know back in their home countries were unfortunately not the same, and they were thus not prepared emotionally for the different experience. Consequently, they did not expect the cultural shock. Besides, other international students who are not from the African continent have come to study and live in Ghana with the expectation of difference and, hence some have arrived in the country prepared for the experience of a challenging new culture. These other international students have read about Ghana and have received some pre-departure orientation before arriving in Ghana. The expectation of difference and prior preparations have equipped the non-African internationals with skills and resources to better deal with acculturative stress. It was discovered in similar research that international students who participated in online social groups and got more informational support from these communities were less likely to feel acculturative stress than their peers who did not use these groups [21].

The communal lifestyles that most African international students have experienced in their home countries, where family, friends, and relatives are always ready to lend a hand in some way, would be another contributing aspect. Since Africans frequently learn their ethical values from people in their efforts to coexist peacefully, it has been noticed that the value orientation tends to discount individual autonomy while supporting community peace and togetherness [56]. As a result, leaving home and losing that sense of family would make them experience a significant sense of loss and homesickness. This is consistent with a study where the authors observed that international students with a social support network in the host country are less likely to be homesick [14]. It is possible that the American or European who underwent the least acculturative stress led a very independent existence and wasn't necessarily experiencing a great deal of familial loss. These go hand in hand with the custom of Ghanaians favouring international students from Europe and the USA, or, in some circumstances, deferring to them.

International students from Asia, Europe, and America with different climatic conditions (cold) found the scorching weather (hot) in Ghana stressful and made their lives difficult. The hot weather made life stressful for the international student from Asia, Europe, America and not their colleagues from Africa since the climatic conditions in most part of Africa tend to be similar. The international students from Asian, Europe, America compared the local conditions in Ghana to what pertains in their home countries where generally the temperate nature of weather in some of their countries of origin is welcoming compared to the hot weather in Ghana. The finding is consistent with a study conducted in the USA among Ecuadorians where they observed that settling into a new environment with adverse weather conditions (cold and rainy) which are extremely different from their place of origin was stressful [15].

Living and learning experiences were perceived as stressful among the international students from Asia, Europe, and America who were interviewed in the case study universities due to the poor internet connections, slow internet speeds, and no notification in the event that classes, events, or activities must be cancelled or rescheduled. The international students interviewed in this study tend to contrast Ghana's living and learning conditions with those in their home nations, that is comparing a less developed country to a developed one where knowledge and information are more widely accessible due to technological advancements.

For several international students from Europe and America surveyed in the two case study universities, false beliefs about their level of income were a significant cause of acculturative stress. Locals often had the erroneous idea about non-African international students, viewing them as rich. Misconceptions about their wealth and influence by local colleagues and the

public, which led to requests for favours, were another source of stress for international students, male or female, who were not from Africa. Acculturative stress was experienced by European, American, and Asian international students, because of their perceived riches and luxury, which led to requests for various favours from the natives. Their African counterparts, on the other hand, were equally stressed because, despite their significant financial contributions to Ghanaian society and the two case study universities, they felt that their contributions were not given the respect they merited and that, as a result, they should have received preferential treatment.

Despite several acculturative stresses found among international students either from Africa, Europe, America, or Asia, the study observed that international students enjoy numerous advantages, including bilingualism, cultural self-awareness, intercultural understanding, and high-status recognition from reputable universities. Learning two languages through English instruction was a significant advantage for many and not as an acculturative stress which is consistent with a study conducted by [57] where the authors argued that native language and bilingualism do not have any impact on acculturative stress.

While making local friends was initially challenging for the international students, those interviewed perceived it to be the main social support avenue that they utilized in dealing with academic and other acculturative stressful experiences. The international students cultivated local friendship networks which helped them in getting the right information regarding classes and how to navigate and understand the academic environment to know what is happening to perform well during class tests, assignments, and examinations and to relate well with the natives in general. The finding was in accordance with other studies where they found that international students participating in social support networks tend to reduce their acculturative stress [1, 2] as well as having local friendship networks [12, 13]. To deal with local language challenges and everyday social, economic, and academic issues, local friendship networks proved helpful for international students.

This paper has contributed to the body of literature on international students in Ghana which is largely non-existent and makes recommendations for training, counselling, and orientation sessions for international students on university campuses. Further, this study provides a deeper understanding of the Acculturative Stress Model [34], from the perspective of international students studying in Ghana. The model posits that besides the changes that occur when people encounter different cultures, cultural and psychological changes have the potential to enhance one's life chances and mental health or virtually destroy one's ability to carry on when one enters a new cultural environment.

Conclusion and recommendations

International students in Ghana face acculturative stress due to rapid price increases for necessities, forced notions of affluence, abrupt changes to academic schedules and classes, inclement weather, and frequent marriage proposals. Western international students being perceived as rich, and wealthy was a major cause of acculturative stress. African international students experienced acculturative stress due to the absence of communal lifestyles as they are accustomed to in their home country. Comparatively European and American international students experienced less acculturative stress due to their independent lifestyles as is generally the case in their countries of origin. Further, being given preferential treatment was a source of stress for international students.

The presence of social support such as friendship networks with nationals of the society, and fellow international students proved to be the major avenues through which international students dealt with acculturative stress experiences. Also, support from local host families and

personal initiatives on the part of the international students themselves helped in dealing with acculturative stress to a certain level. This paper makes the following recommendations for international student affairs offices and exchange programs on Ghanaian university campuses: they should offer staff training for faculty members and university employees, as well as customize counselling and orientation services to meet the needs of the varied populations of international students.

Despite the several stressors reported by the international students that might not be exhaustive, since they may have underestimated negative experiences due to bias towards social desire and subjective stress perception, leading to inconsistencies in self-reporting. The public and private institutions used in this analysis may be a limitation since institutional support systems differ across universities, which may have an impact on the acculturative stress levels of international students, and these variations may have some effect on the study findings. The methodological technique adopted could be a significant drawback, as the findings may not apply to all international students other than those who participated in this study.

Author Contributions

Conceptualization: Kwasi-Gyasi Gyamerah, Prof Joseph Osafo, Angela Anarfi Gyasi-Gyamerah, Evans Sakyi Boadu.

Methodology: Kwasi-Gyasi Gyamerah, Evans Sakyi Boadu.

Supervision: Prof Joseph Osafo.

Writing – original draft: Kwasi-Gyasi Gyamerah, Evans Sakyi Boadu.

Writing – review & editing: Kwasi-Gyasi Gyamerah, Prof Joseph Osafo, Angela Anarfi Gyasi-Gyamerah, Evans Sakyi Boadu.

References

1. Lashari SA, Awang-hashim R, Lashari TA, Kaur A. Acculturation stress and social support for international students ' adjustment in Malaysia: does language proficiency matter? J Appl Res High Educ. 2022; 15(2):496–508.
2. Sullivan C, Kashubeck-West S. The Interplay of International Students ' Acculturative Stress, Social Support, and Acculturation Modes. J Int Students. 2015; 5(1):1–11.
3. Smith RA, Khawaja NG. International Journal of Intercultural Relations A review of the acculturation experiences of international students. Int J Intercult Relations [Internet]. 2011; 35(6):699–713. Available from: https://doi.org/10.1016/j.ijintrel.2011.08.004
4. Berry JW. Mutual attitudes among immigrants and ethnocultural groups in Canada. Int J Intercult Relations. 2006; 30:719–34.
5. Berry JW. Acculturation: Living successfully in two cultures. Int J Intercult Relations. 2005; 29:697–712.
6. Lee JS, Koeske GF, Sales E. Social support buffering of acculturative stress: a study of mental health symptoms among Korean international students. Int J Intercult Relations. 2004; 28(5):399–414.
7. Väfors FM, Chin D, DeMarinis V. Stressors, anxiety, acculturation and adjustment among international and North American students. Int J Intercult Relations. 2008; 32(3):244–59.
8. Poyrazli S, Kavanaugh PR, Baker A, At-Timimi N. Social Support and Demographic Correlates of Acculturative Stress in International Students. J Coll Couns. 2004; 7:73–82.
9. Hansen HR, Shneyderman Y, McNamara GS, Grace L. Assessing Acculturative Stress of International Students at a U. S. Community College. J Int Students. 2018; 8(1):215–32.
10. Kristiana IF, Karyanta NA, Simanjuntak E, Prihatsanti U, Ingarianti TM. Social Support and Acculturative Stress of International Students. 2022;
11. Fedolina BF, Saptandari EW. Perceived Social Support as Predictor of Acculturative Stress Among Indonesian Exchange Students in Europe. J Psikol Integr. 2024; 12(1):83–101.
12. Chidinma Chikwe, Oriana. Piskorz-Ryń SAS. Cultural Adaptation Stress and Social Competence as Determinants of Hopelessness. J Psychosociological Res Fam Cult. 2024;2(3):1–10.

13. Genkova P, Schreiber H. Interculturality and social skills? Relationships of the stays abroad of students of different faculties with intercultural and social competence. J Community Appl Soc Psychol. 2021; 31:410–24.

14. Hendrickson B, Rosen D, Aune RK. An analysis of friendship networks, social connectedness, homesickness, and satisfaction levels of international students. Int J Intercult Relations [Internet]. 2011; 35 (3):281–95. Available from: https://doi.org/10.1016/j.ijintrel.2010.08.001

15. Baeza-Rivera MJ, Salazar-Fernández C, Manríquez-Robles D, Salinas-Oñate N, Smith-Castro V. Acculturative Stress, Perceived Social Support, and Mental Health: The Mediating Effect of Negative Emotions Associated with Discrimination. Int J Environ Res Public Heal. 2022; 19:1–17. https://doi.org/10.3390/ijerph192416522 PMID: 36554406

16. Bae SM. The relationship between social capital, acculturative stress and depressive symptoms in multicultural adolescents: Veri fi cation using multivariate latent growth modeling. Int J Intercult Relations. 2020; 74:127–35.

17. Li L, Peng W. Transitioning through social media: International students' SNS use, perceived social support, and acculturative stress. Comput Human Behav [Internet]. 2019; 98:69–79. Available from: https://doi.org/10.1016/j.chb.2019.03.011

18. Wang P, Chen C, Yim IS. Does perceived support moderate the link between acculturative stress and problematic eating behaviors? The role of family, significant other, and friend support. Int J Eat Disord. 2023; 56(4):1–12. https://doi.org/10.1002/eat.23941 PMID: 36942361

19. Ra Y an. Roles of social support on acculturative stress of Asian international students. Curr Psychol. 2024;43:3882–8.

20. Cheng M, Friesen A, Adekola O. Using emotion regulation to cope with challenges: a study of Chinese students in the United Kingdom. Cambridge J Educ [Internet]. 2018; 49(2):133–45. Available from: http://doi.org/10.1080/0305764X.2018.1472744

21. Ye J. An Examination of Acculturative Stress, Interpersonal Social Support, and Use of Online Ethnic Social Groups among Chinese International Students An Examination of Acculturative Stress, Interpersonal Social Support, and Use Chinese International Stude. Howard J Commun ISSN. 2007; 17(1):1–20.

22. Scheaffer RL, Mendenhall W, Ott L. Elementary Survey Sampling. Third edition. PWK-Kent Publishing Co., Boston, MA, USA. 1986.

23. Lee J yeon, Ciftci A. Asian international students ' socio-cultural adaptation: Influence of multicultural personality, assertiveness, academic self-efficacy, and social support. Int J Intercult Relations [Internet]. 2014;38:97–105. Available from: https://doi.org/10.1016/j.ijintrel.2013.08.009

24. Eustace RW (2007). Factors influence acculturative stress among international students. (Unpublished doctoral dissertation). Kansas State University, Missouri. 2007.

25. Thomas M, Choi Jb. Acculturative Stress and Social Support among Korean and Indian Immigrant Adolescents in the United States Department of Social Work. J Sociol Soc Welf. 2006; 33(2):123–44.

26. Swagler MA, Ellis M V. Crossing the Distance: Adjustment of Taiwanese Graduate Students in the United States. J Couns Psychol. 2003; 50(4):420–37.

27. Akhtar M, Kröner-herwig B. Acculturative Stress Among International Students in Context of Socio-Demographic Variables and Coping Styles. Curr Psychol. 2015; 34:803–15.

28. Eland AJ. Intersection of academics and culture: The academic experience of International graduate students. Diss Abstr Int. 2001; 62(03).

29. Sanchez M, Dillon F, Ruffin B, Rosa MD La. The Influence of Religious Coping on the Acculturative Stress of Recent Latino Immigrants. J Ethn Cult Divers Soc Work ISSN. 2012; 21(3):171–94. https://doi.org/10.1080/15313204.2012.700443 PMID: 24307865

30. Bowen TL, Morgan R. Stress, Spirituality and Health Promoting Behaviors Among African American College Students. West J Black Stud. 2004; 28(I):283–92.

31. Holt CL, Lewellyn L, Rathweg MJ. Exploring Religion–Health Mediators among African American Parishioners. J Health Psychol. 2005; 10(4):511–27. https://doi.org/10.1177/1359105305053416 PMID: 16014389

32. Ellison CG, Finch BK, Ryan DN, Salinas JJ. Religious Involvement And Mexican-Origin Adults In California. J Community Psychol. 2009; 37(2):171–93.

33. Yu B, Chen X, Li S, Liu Y, Jacques-tiura AJ, Yan H. Acculturative Stress and Influential Factors among International Students in China: A Structural Dynamic Perspective. PLoS One. 2014; 9(4).

34. Berry AJW, Kim U, Minde T, Mok D, Minde T, Mok D. Comparative Studies of Acculturative Stress. Int Migr Rev. 1987; 21(3):491–511.

35. Williams C. L., & Berry JW. Primary prevention of acculturative stress among refugees: Application of psychological theory and practice. Am Psychol. 1991; 46(6):632–41.

36. Sam DL, Berry JW. Acculturation: When Individuals and Groups of Different Cultural Backgrounds Meet. Perspect Psychol Sci. 2010; 5(4):472–81. https://doi.org/10.1177/1745691610373075 PMID: 26162193
37. Smith JA, Flowers P, Larkin M. Interpretative phenomenological analysis: Theory, method and research. London: Sage. Vol. 2nd Editio. 2009.
38. Etikan I, Musa SA, Alkassim RS. Comparison of Convenience Sampling and Purposive Sampling. Am J Theor Appl Stat. 2016; 5(1):1–5.
39. Emerson RW. Convenience Sampling, Random Sampling, and Snowball Sampling: How Does Sampling Affect the Validity of Research? J ofVisual Impair Blind. 2015;(April):164–8.
40. Lucas SR. Beyond the existence proof: ontological conditions, epistemological implications, and in-depth interview research. Qual Quant. 2014; 48:387–408.
41. Nguyen TQT. Conducting semi-structured interviews with the Vietnamese. Qual Res J. 2015; 15(1):35–46.
42. Bowen G. Document Analysis as a Qualitative Research Method. 2017;(October).
43. Slevin E, Sines D. Enhancing the truthfulness consistency and transferability of a qualitative study. Nurse Res. 2000; 7(2):79–97.
44. Alhojailan MI, Ibrahim M. Thematic Analysis: A Critical Review of Its Process and Evaluation. WEI Int Eur Acad Proc. 2012; 1(2011):8–21.
45. Clarke V, Braun V. Thematic analysis. J Posit Psychol [Internet]. 2017; 9760:1–2. Available from: https://doi.org/10.1080/17439760.2016.1262613
46. Smith J, Osborn M. Interpretive phenomenological analysis. In: In JA Smith (Ed) Qualitative psychology: A practical guide to research methods (pp51-80)London: Sage. 2003. p. 51–50.
47. Berg BL. Qualitative Research Methods for the Social Sciences (Fourth Edition). Boston: Allyn and Bacon. 2001.
48. Bowen GA. Document Analysis as a Qualitative Research Method. Qual Res J. 2009; 9(2):27–40.
49. Campbell R, Goodman-williams R, Feeney H, Fehler-cabral G. Assessing Triangulation Across Methodologies, Methods, and Stakeholder Groups: The Joys, Woes, and Politics of Interpreting Convergent and Divergent Data. Am J Eval. 2020; 41(1):125–44.
50. Turner SF, Cardinal LB, Burton RM. Research Design for Mixed Methods: A Triangulation-based Framework and Roadmap. Organ Res Methods. 2017; 20(2):243–67.
51. Lincoln YS, Guba EG. But Is It Rigorous? Authenticity in Trustworthiness and Naturalistic Evaluation. New Dir Progr Eval. 1986;(30):73–84.
52. Baker S, Waterfield J, Bartlam B. Saturation in qualitative research: exploring its conceptualization and operationalization. Qual Quant. 2018; 52(4):1893–907. https://doi.org/10.1007/s11135-017-0574-8 PMID: 29937585
53. Ghana Statistical Service. Ghana, 2010 Population and Housing Census. Summary Report of Final Results. Ghana Statistical Service, May 2012. 2012.
54. Riaz MN, Nawaz S. Predictors of acculturative stress in Chinese academic sojourners in Pakistan. Rawal Med J. 2020; 45(4):902–5.
55. Yeh CJ, Inose M. International students ' reported English fluency, social support satisfaction, and social stress International students ' reported English fluency, social support satisfaction, and social connectedness as predictors of acculturative stress. Couns Psychol Q. 2010; 16(1):15–28.
56. Gyekye K. African Cultural Values: An Introduction. Accra: Sankofa Publishing Company. Vol. 28. 2003.
57. Lueck K, Wilson M. Acculturative stress in Asian immigrants: The impact of social and linguistic factors. Int J Intercult Relations. 2010; 34:47–57.
